# Clinical efficacy of probiotics in the treatment of diabetes kidney disease: a systematic review and meta-analysis

**DOI:** 10.3389/fmicb.2025.1729409

**Published:** 2026-01-12

**Authors:** Liangjing Liu, Haolan Liu, Xiaoling Mao, Chao Li, Weitong Yan, Zhongmin Ou, Xianhui Li

**Affiliations:** School of Medicine, Jishou University, Jishou, Hunan, China

**Keywords:** clinical efficacy, diabetes kidney disease, meta-analysis, probiotic, systematic review

## Abstract

**Objective:**

Diabetic kidney disease (DKD) is a major microvascular complication of diabetes and the leading cause of end-stage renal disease. Growing evidence suggests that gut microbiota dysbiosis may contribute to DKD progression, and probiotics have been proposed as a potential adjunctive therapy. However, existing clinical findings remain inconsistent, with some trials reporting benefits in blood urea nitrogen (BUN), estimated glomerular filtration rate (eGFR), and glycosylated hemoglobin (HbA1c), while others show no significant improvements. This study aimed to systematically evaluate the clinical efficacy of probiotics in patients with DKD.

**Methods:**

Relevant randomized controlled trials (RCTs) were identified through PubMed, Embase, Web of Science, the Cochrane Library, and the Chinese Science Citation Database up to October 2025. Data were synthesized using RevMan 5.3 and Stata 16.0. The risk of bias was assessed using the Cochrane Risk of Bias tool version 1.0 (RoB 1.0), publication bias was evaluated with Egger’s test, and the certainty of evidence was graded according to the GRADE approach.

**Results:**

Seven RCTs involving 502 participants were included. Meta-analysis showed that probiotics significantly reduced serum creatinine [mean difference (MD) −0.09 mg/dL, 95% confidence interval (CI) −0.14 to −0.04], BUN (MD −1.58 mg/dL, 95% CI −2.80 to −0.36), fasting blood glucose (MD −0.48 mmol/L, 95% CI −0.89 to −0.07), triglycerides (MD −19.17 mg/dL, 95% CI −35.14 to −3.20), total cholesterol (MD −11.68 mg/dL, 95% CI −20.37 to −2.99), low-density lipoprotein cholesterol (MD −12.72 mg/dL, 95% CI −18.76 to −6.67), high-sensitivity C-reactive protein (MD −1.59 mg/L, 95% CI −2.31 to −0.88), and malondialdehyde (MD −0.52 μmol/L, 95% CI −0.91 to −0.13). No significant effects were observed on 24-h urine protein, eGFR, 2-h postprandial blood glucose, HbA1c, insulin, high-density lipoprotein cholesterol, or total antioxidant capacity. Egger’s test indicated no significant publication bias for most outcomes, except for potential bias in eGFR. The certainty of evidence ranged from moderate to very low, and the strength of recommendation was strong.

**Conclusion:**

Probiotic supplementation may improve renal function, glycemic control, lipid metabolism, and inflammation/oxidative stress in DKD patients. Further large, high-quality RCTs are warranted to confirm these findings.

## 1 Introduction

Diabetic kidney disease (DKD) is a chronic kidney disorder caused by diabetes, characterized by persistent proteinuria with or without a decline in glomerular filtration rate (GFR) (Liu et al., 2023). It represents one of the most common and severe microvascular complications of diabetes and is the leading cause of end-stage renal disease (ESRD) worldwide (Gupta et al., 2023). Epidemiological data indicate that in 2024, the global population of adults with diabetes reached 588.7 million (International Diabetes Federation, 2025), among whom approximately 20–50% may progressively develop DKD (Uma et al., 2025). DKD not only leads to gradual renal failure but also markedly increases the risk of cardiovascular events, making it one of the main causes of mortality among patients with diabetes (Watanabe et al., 2009; Di Marco et al., 2023; Bhagwat and Kumar, 2023).

**Figure 1:**
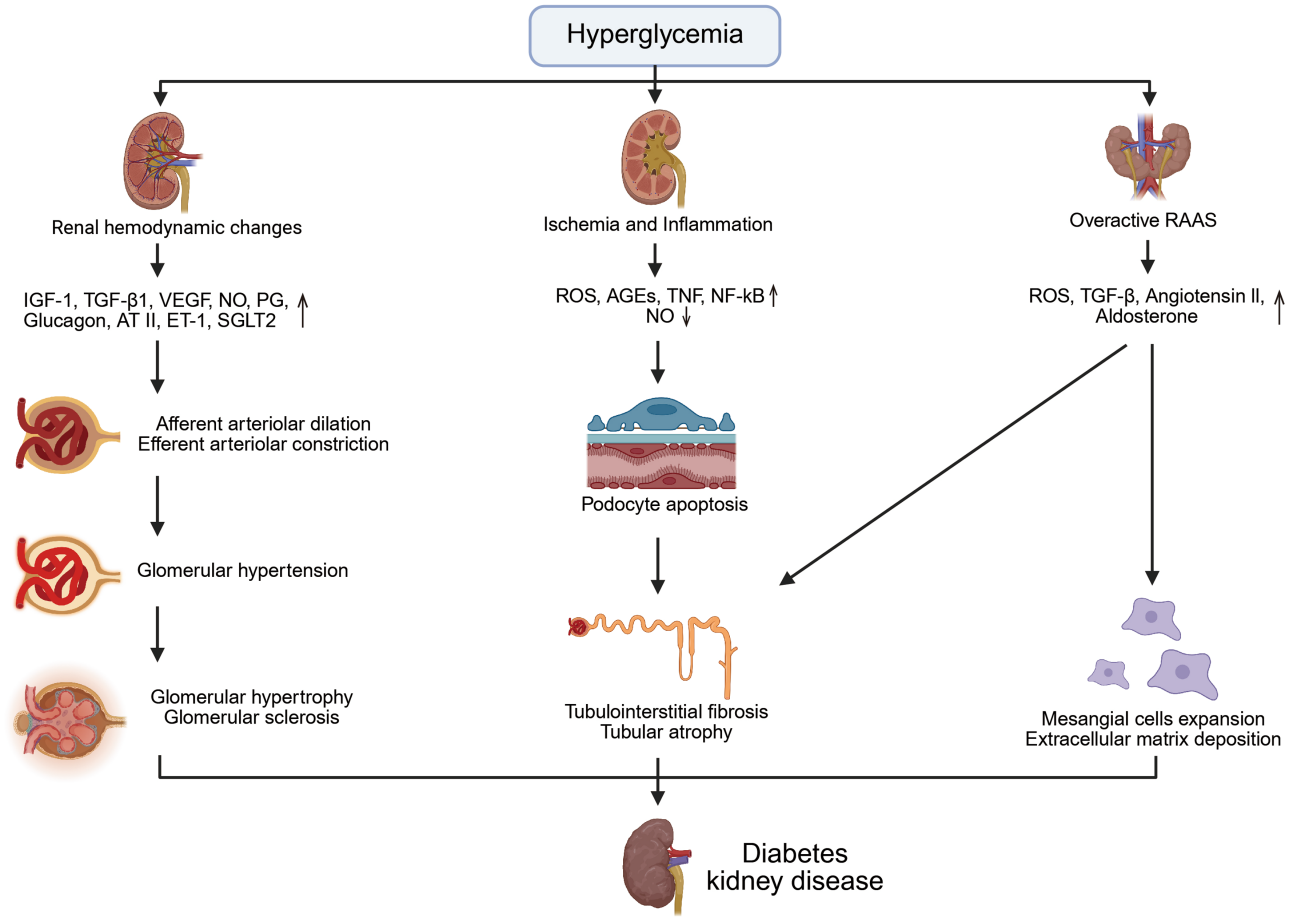
Pathophysiology of diabetes kidney. IGF-1, insulin-like growth factor 1; TGF-β1, transforming growth factor β1; VEGF, vascular endothelial growth factor; NO, nitric oxide; PG, prostaglandin; AT II, angiotensin II; ET-1, endothelin-1; SGLT2, sodium glucose co-transporters 2; ROS, reactive oxygen species; AGEs, advanced glycation end products; TNF, tumor necrosis factor; NF-κB, nuclear factor kappa-light-chain-enhancer of activated B cells; RAAS, renin-angiotensin-aldosterone system.

Chronic and persistent hyperglycemia is the fundamental driving force underlying the onset and progression of DKD, driving hemodynamic alterations, ischemia- and inflammation-related injury, and activation of the renin–angiotensin–aldosterone system (RAAS) (Wu et al., 2025). Sustained elevations in glucose trigger early hemodynamic disturbances by altering the expression of multiple vasoactive and growth-related mediators, including insulin-like growth factor-1 (IGF-1), transforming growth factor-β1 (TGF-β1), vascular endothelial growth factor (VEGF), nitric oxide (NO), prostaglandins (PG), glucagon, angiotensin II (Ang II), endothelin-1 (ET-1), and sodium–glucose cotransporter-2 (SGLT2) (Elian et al., 2025; Sanglard et al., 2025; Dwivedi and Sikarwar, 2025). These changes lead to afferent arteriolar dilation and efferent arteriolar constriction, resulting in glomerular hyperperfusion, increased filtration, and elevated intraglomerular pressure (Ratan et al., 2025). Prolonged exposure to these abnormalities causes glomerular hypertrophy, mesangial expansion, and thickening of the glomerular basement membrane (Ratan et al., 2025). Moreover, hyperglycemia induces renal ischemia and activates inflammatory and oxidative pathways central to DKD progression (Liu et al., 2025). Excess glucose increases reactive oxygen species (ROS) production, accelerates advanced glycation end products (AGEs) accumulation, and activates nuclear factor kappa-light-chain-enhancer of activated B cells (NF-κB) signaling, thereby reducing NO bioavailability and exacerbating endothelial dysfunction and hypoxia (Aguilera-Martínez et al., 2025; Hou et al., 2025). These metabolic insults contribute to podocyte apoptosis, disruption of the glomerular filtration barrier, and tubular epithelial injury, ultimately promoting tubulointerstitial fibrosis (Aguilera-Martínez et al., 2025). Furthermore, hyperglycemia also enhances systemic and intrarenal RAAS activation (Sanglard et al., 2025). Elevated Ang II further increases oxidative stress, stimulates TGF-β expression, and promotes aldosterone release, accelerating mesangial matrix accumulation, fibrosis, and tubular injury (Dwivedi and Sikarwar, 2025; Apte et al., 2024). Collectively, these hemodynamic, metabolic, inflammatory, and hormonal disturbances interact in a self-reinforcing manner, driving glomerular hypertension, podocyte loss, tubular damage, and progressive fibrosis, as illustrated in Figure 1.

Current therapeutic strategies for DKD focus on comprehensive risk factor control. Standard interventions include strict glycemic control, blood pressure management, lipid regulation, and inhibition of RAAS using angiotensin-converting enzyme inhibitors or Ang II receptor blockers (Młynarska et al., 2024; Han et al., 2024; Gaddy et al., 2025). Recent therapeutic advances, such as SGLT2 inhibitors and glucagon-like peptide-1 (GLP-1) receptor agonists, have demonstrated renoprotective benefits by reducing albuminuria and delaying estimated GFR (eGFR) decline (Agarwal et al., 2025; Joseph, 2025). Despite these advancements, significant limitations remain. For one, a proportion of patients continue to experience progressive proteinuria and decline in renal function even under optimized therapy, including newer renoprotective agents such as SGLT2 inhibitors and GLP-1 receptor agonists (Vozza et al., 2025). For another, the use of antidiabetic medications is often constrained by their adverse effects and safety concerns. Metformin may cause gastrointestinal intolerance and, in rare cases, lactic acidosis (Yao et al., 2025); sulfonylureas are associated with hypoglycemia and weight gain (Li and Yun, 2025); and thiazolidinediones may induce weight gain, edema, and increase the risk of heart failure or fractures (Natale et al., 2025). In addition, SGLT2 inhibitors such as empagliflozin and dapagliflozin may cause genital infections or volume depletion (Joseph, 2025). Meanwhile, GLP-1 receptor agonists, such as liraglutide and semaglutide, are frequently associated with gastrointestinal intolerance and remain relatively costly, which further limits their accessibility (Uma et al., 2025). Therefore, there is a growing need for safe, effective, and affordable adjunctive strategies to further improve clinical outcomes in DKD.

With advances in microbiology, increasing attention has been directed toward the relationship between gut microbiota dysbiosis and DKD (Tian et al., 2023). A metagenomic study revealed that compared with healthy individuals, patients with DKD exhibit a significant reduction in the abundance of beneficial bacteria such as *Clostridium*, *Eubacterium*, *Roseburia intestinalis*, *Lachnospira*, and *Intestinibacter*, while *Bacteroides stercoris* is markedly enriched (Zhang et al., 2022). Notably, a specific, as-yet-unnamed strain of *Clostridium*, referred to as *Clostridium* sp. *26_22* in metagenomic studies, has been reported to show a negative correlation with serum creatinine levels (Zhang et al., 2022). Based on these findings, some researchers have proposed that probiotic supplementation and restoration of gut microbiota homeostasis may represent a potential therapeutic approach for DKD (Jiang et al., 2021; Cai et al., 2024; Mafi et al., 2018).

Clinical trials have demonstrated that 12-week of probiotic supplementation significantly reduced fasting blood glucose (FBG), serum insulin, triglycerides (TG), malondialdehyde, and advanced glycation end-products in patients with DKD, while increasing the quantitative insulin sensitivity check index and plasma total glutathione levels (Mafi et al., 2018). In a meta-analysis, Tarrahi et al. (2022) reported that probiotics significantly reduced FBG and serum creatinine (SCR) levels in DKD patients but showed no significant effects on glycated hemoglobin (HbA1c), insulin, blood urea nitrogen (BUN), or eGFR. In contrast, another meta-analysis conducted by Dai et al. (2022) found that probiotics not only lowered FBG and HbA1c levels but also reduced SCR and BUN levels. These findings indicate that existing evidence remains inconsistent: while most studies consistently show reductions in FBG and SCR, the effects on HbA1c, insulin, BUN, and eGFR differ markedly across trials, leading to ongoing uncertainty regarding the overall metabolic and renal benefits of probiotics in DKD. It is noteworthy that both Tarrahi et al. (2022) and Dai et al. (2022) included a clinical trial involving uremic patients, which may have introduced additional confounding factors. Furthermore, Tarrahi et al. (2022) only included studies published up to 2019, resulting in temporal limitations. Considering these confounding effects, time constraints, and contradictory findings, current evidence remains insufficient to draw robust conclusions regarding the effects of probiotics on patients with DKD.

In light of the unresolved inconsistencies and methodological shortcomings of previous studies, there is a pressing need to re-evaluate the evidence. This re-evaluation should involve more stringent inclusion criteria, clearer population definitions, and a broader range of clinically relevant outcomes. Addressing these gaps is crucial for determining whether probiotics have meaningful effects on metabolic, renal, inflammatory, and oxidative pathways. Therefore, by reassessing the available trials with improved methodological rigor, this study aims to provide a more reliable and up-to-date evidence base to inform clinical decision-making and guide future research.

## 2 Methods

This meta-analysis was performed following the guidelines established by the Preferred Reporting Items for Systematic Reviews and Meta-Analyses (PRISMA) (Page et al., 2021).

### 2.1 Inclusion and exclusion criteria

Studies were included if they met the following criteria: (i) Participants: adults (≥18 years) diagnosed with DKD based on established clinical or laboratory criteria, including persistent albuminuria or proteinuria (≥30 mg/24 h or urine albumin-to-creatinine ratio ≥30 mg/g) and/or reduced eGFR (<60 mL/min/1.73 m^2^) (de Boer et al., 2022); (ii) Intervention: probiotics combination with standard treatment; (iii) Comparison: standard treatment alone. (iv) Outcomes: at least one of the following was reported—renal function indicators [SCR, 24-h urine protein (24 h UP), eGFR, BUN], blood glucose levels [FBG, 2-h postprandial blood glucose (2 h PBG), HbA1c, insulin], blood lipid levels [TG, total cholesterol (TC), low-density lipoprotein cholesterol (LDL-C), high-density lipoprotein cholesterol (HDL-C)], and inflammatory and oxidative markers [high-sensitivity C-reactive protein (hs-CRP), total antioxidant capacity (TAC), malondialdehyde (MDA)]; (v) Study design: randomized controlled trials (RCTs).

Exclusion criteria were: (i) Duplicate publications; (ii) Studies with insufficient data to extract or analyze relevant outcomes; (iii) Participants diagnosed with uremia or ESRD.

### 2.2 Literature search strategy

A comprehensive search was performed in PubMed, Embase, Web of Science, the Cochrane Library, and Chinese Science Citation Database (CSCD) from their inception to October 1, 2025. The search fields are Topic or Title/Abstract, and the search query is as follows: (Probiotic OR Probiotics OR Synbiotic OR Synbiotics OR Bifidobacterium OR Bifidobacteria OR *Bacillus bifida* OR Yeast OR *Saccharomyces cerevisiae* OR *Saccharomyces italicus* OR *Saccharomyces oviformis* OR *S. cerevisiae* OR *S. cerevisiae* OR *Saccharomyces uvarum* var. *melibiosus* OR *Candida robusta* OR *Saccharomyces capensis* OR *Lactobacillus acidophilus* OR *Lactobacillus amylovorus* OR Lactobacill OR Lactic acid bacteria OR *Clostridium butyricum* OR Bacillus OR Natto Bacteria OR Streptococcus thermophiles OR Enterococcus) AND (Diabetic nephropathy OR Diabetic nphropathies OR Diabetic Kidney Disease OR Diabetic Kidney Diseases OR Diabetic Glomerulosclerosis OR Intracapillary Glomerulosclerosis OR Kimmelstiel Wilson Disease OR Nodular Glomerulosclerosis OR Kimmelstiel Wilson Syndrome). No restrictions were applied to language or publication status. Reference lists of relevant reviews and included studies were manually screened to identify additional eligible articles.

### 2.3 Study selection process

A systematic approach combining a reference management tool and manual evaluation was employed to screen the literature (Yu et al., 2023). All retrieved records were imported into EndNote for deduplication. Two reviewers independently screened the titles and abstracts for potential eligibility, followed by full-text assessment of the remaining articles. Discrepancies were resolved by discussion or consultation with a third reviewer. The study selection process was documented in a PRISMA flow diagram.

### 2.4 Data extraction

Two reviewers independently extracted data using a pre-designed data collection form (Yu et al., 2024). Extracted information included: (i) basic study characteristics (first author, year of publication, country, study design); (ii) participant characteristics (sample size, age, sex distribution, weight, disease duration); (iii) intervention details (strain of probiotics, dosage, frequency, duration); (iv) funding sources and potential conflicts of interest. Any disagreements were resolved by discussion or adjudication by a third reviewer.

### 2.5 Risk of bias assessment

The risk of bias of included RCTs was independently evaluated by two reviewers using the Cochrane Collaboration’s Risk of Bias tool (RoB 1.0) (Higgins et al., 2011). The following seven domains were assessed: random sequence generation, allocation concealment, blinding of participants and personnel, blinding of outcome assessment, incomplete outcome data, selective outcome reporting, and other sources of bias. Each domain was judged as “low risk,” “high risk,” or “unclear risk” of bias. Disagreements between reviewers were resolved by discussion or, if necessary, consultation with a third reviewer.

### 2.6 Statistical analysis

Meta-analysis was conducted using RevMan (version 5.3) and Stata (version 16.0). Continuous outcomes were expressed as mean differences (MDs) or standardized mean differences (SMDs) with 95% confidence intervals (CIs). Dichotomous outcomes were presented as risk ratios (RRs) with 95% CIs (Li et al., 2025). Statistical heterogeneity among studies was assessed using the *I*^2^ statistic. An *I*^2^ > 50% indicated substantial heterogeneity, in which case a random-effects model was applied; otherwise, a fixed-effects model was used (Migliavaca et al., 2022). *p* < 0.05 was considered statistically significant.

For outcomes with *I*^2^ > 50%, leave-one-out sensitivity analysis was performed, whereby each study was sequentially omitted to investigate potential sources of heterogeneity and assess the robustness of the pooled results. The stability of the results was judged by observing whether the direction and magnitude of the overall effect size changed materially after the exclusion of any single study. If the pooled effect estimates remained consistent, the results were considered robust (Patsopoulos et al., 2008). Additionally, when the number of included studies for outcomes with high heterogeneity was five or more, subgroup analyses were performed based on factors such as gender, mean age, body weight, HbA1c, SCR, disease duration, probiotic preparations, and treatment duration. These subgroup analyses aimed to investigate the heterogeneity originating from clinical aspects and assess the robustness of the results (Richardson et al., 2019).

### 2.7 Publication bias

Publication bias was assessed using funnel plot symmetry when more than 10 studies were included in a meta-analysis (Jin et al., 2015). When fewer than 10 studies were available, funnel plot inspection was not performed due to limited precision; instead, Egger’s regression test was applied to quantitatively evaluate potential publication bias (Lin and Chu, 2018). A *p*-value greater than 0.05 in Egger’s test was considered to indicate no potential publication bias.

### 2.8 Certainty of evidence

The certainty of evidence for each outcome was assessed using the Grading of Recommendations Assessment, Development and Evaluation (GRADE) approach (Prasad, 2024). The certainty of evidence was classified into four levels: high, moderate, low, or very low, based on risk of bias, inconsistency, indirectness, imprecision, and publication bias.

## 3 Results

### 3.1 Study selection

**Figure 2:**
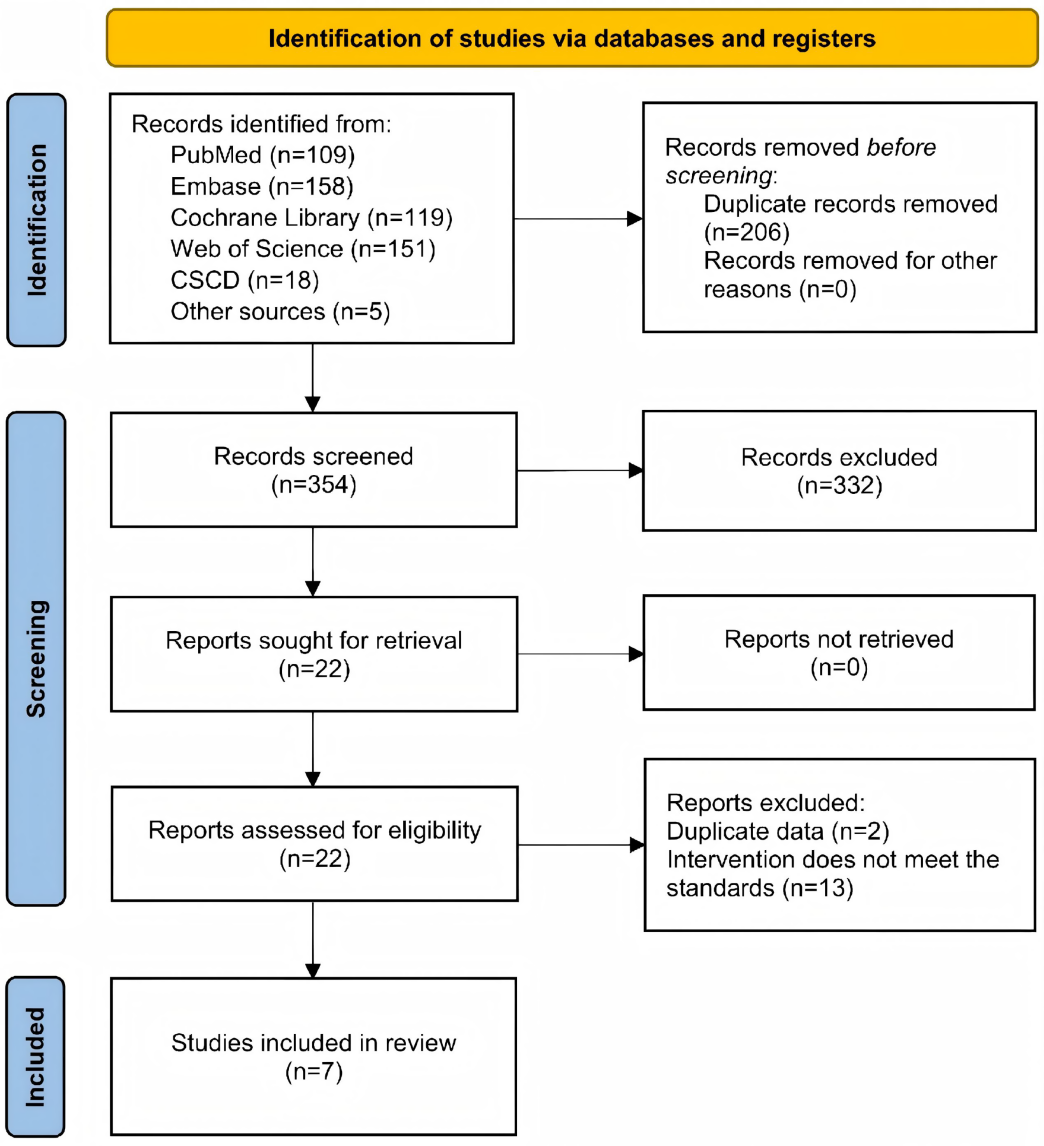
PRISMA flowchart.

A total of 560 records were identified through the electronic databases, including PubMed (*n* = 109), Embase (*n* = 158), Cochrane Library (*n* = 119), Web of Science (*n* = 151), and CSCD (*n* = 18). An additional 5 studies were identified from other sources. After removing 206 duplicates, 354 records remained. Screening of titles and abstracts excluded 332 articles, and 22 full-text articles were assessed for eligibility. Among these, 15 articles were excluded (2 for reporting duplicate data and 13 for not meeting the intervention criteria). Finally, 7 RCTs (Jiang et al., 2021; Mafi et al., 2018; Abbasi et al., 2018; Mazruei Arani et al., 2019; Firouzi et al., 2015; Miraghajani et al., 2017; Tang et al., 2020) were included in the meta-analysis. The study selection process is summarized in the PRISMA flow diagram (Figure 2).

### 3.2 Basic characteristics of included studies

A total of 7 randomized controlled trials involving 502 participants were included (Jiang et al., 2021; Mafi et al., 2018; Abbasi et al., 2018; Mazruei Arani et al., 2019; Firouzi et al., 2015; Miraghajani et al., 2017; Tang et al., 2020). Among them, 255 participants received probiotics in addition to standard treatment, while 247 participants received standard treatment alone. Overall, the mean proportion of female participants was 52.0%, with a mean age of 56.4 years and an average body weight of 74.1 kg. Baseline HbA1c levels ranged from 6.8 to 7.9 mmol/L, SCR from 0.8 to 3.3 mg/dL, and disease duration from 6.9 to 9.3 years. Three studies administered single-strain probiotics, whereas four studies used multi-strain probiotic formulations. The intervention duration ranged between 8 and 12 months. Detailed characteristics of the included studies are presented in Table 1.

**Table 1:** Basic characteristics of included studies.

Study ID	Country	Sample	Female (%)	Age (years)	Weight (kg)	HbA1c (%)	SCR (mg/dL)	Disease duration (years)	Intervention	Probiotics	Treatment duration (weeks)
Abbasi et al. (2018)	Iran	20	55.0	56.9	70.8	—	1.0	8.70	Probiotic soy milk 200 mL qd	*L. plantarum* A7	8
		20	45.0	53.6	71.6	—	1.0	6.90	Conventional soy milk 200 mL qd	—	8
Mazruei Arani et al. (2019)	Iran	30	—	62.7	79.6	—	1.6	—	Probiotic honey 25 g qd	*B. coagulans* T4	12
		30	—	60.3	78.0	—	1.3	—	Honey 25 g qd	—	12
Firouzi et al. (2015)	Malaysia	68	45.6	52.9	74.6	—	0.8	—	Probiotic preparation	*L. acidophilus*, *L. casei*, *L. lactis*, *B. bifidum*, *B. longum*, *B. infantis*	12
		68	50	54.2	76.6	—	0.9	—	Placebo preparation	—	12
Jiang et al. (2021)	China	42	64.3	56.0	—	7.3	—	—	Probiotic preparation	*B. bifidum*, *L. acidophilus*, *S. thermophilus*	12
		34	64.7	56.1	—	7.9	—	—	Placebo preparation	—	12
Mafi et al. (2018)	Iran	30	—	58.9	69.3	6.8	1.3	—	Probiotic preparation	*L. acidophilus*, *B. bifidum*, *L. reuteri*, *L. fermentum*	12
		30	—	60.9	70.5	6.9	1.4	—	Placebo preparation	—	12
Miraghajani et al. (2017)	Iran	20	—	56.9	70.8	—	—	8.70	Probiotic soy milk 200 mL qd	*L. plantarum* A7	8
		20	—	53.6	71.6	—	—	6.90	Conventional soy milk 200 mL qd	—	8
Tang et al. (2020)	China	45	51.1	55.8	—	6.9	3.0	9.30	Probiotic preparation	*B. longum*, *L. bulgaricus*, *S. thermophilus*	12
		45	46.5	56.9	—	7.3	3.3	9.40	None	—	12

### 3.3 Risk of bias

**Figure 3:**
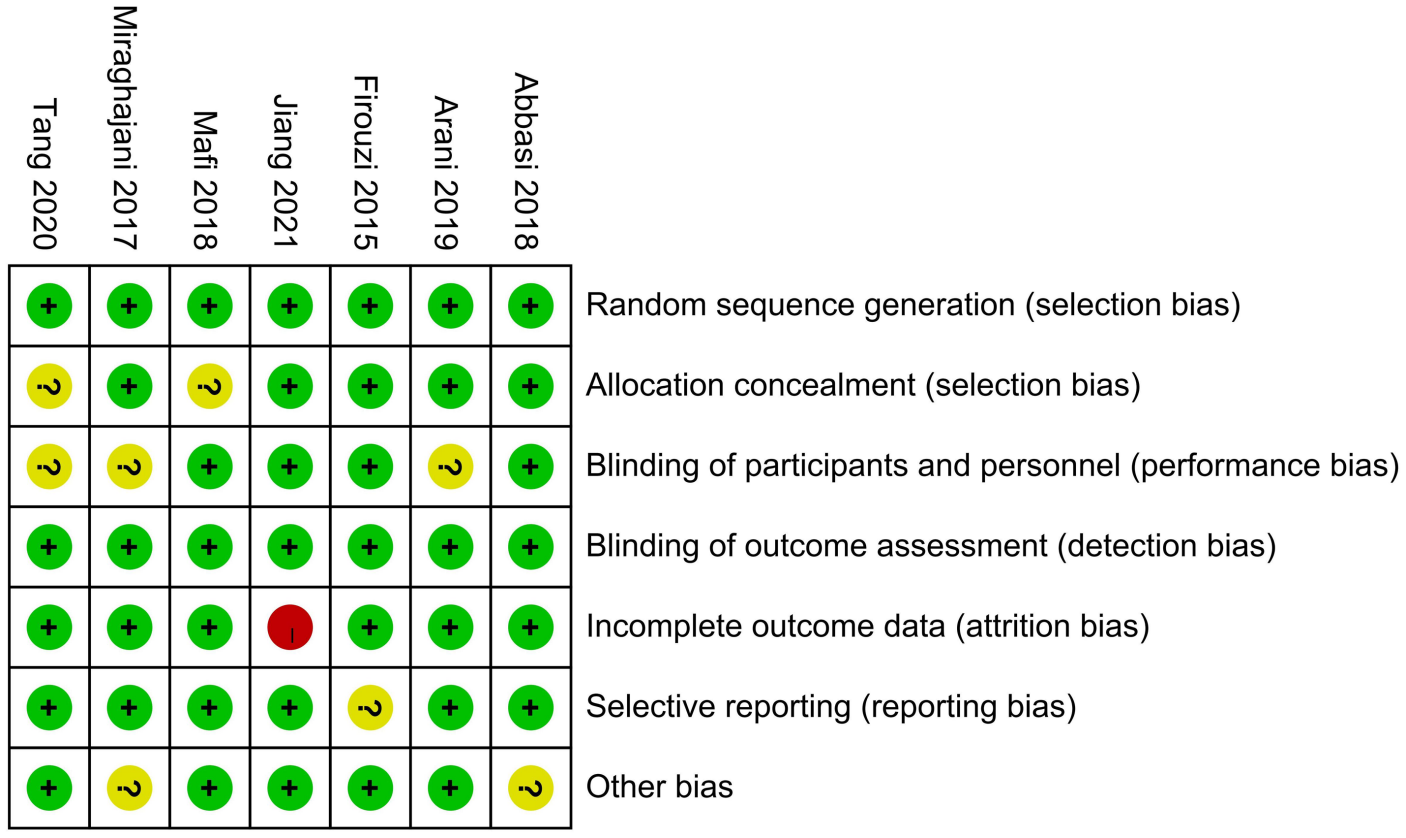
Risk assessment of bias.

According to the RoB 1.0 assessment (Figure 3), allocation concealment was rated as having an unclear risk in the studies conducted by Mazruei Arani et al. (2019) and Tang et al. (2020); blinding of participants and personnel was rated as having an unclear risk in the studies conducted by Mazruei Arani et al. (2019), Miraghajani et al. (2017), and Tang et al. (2020); incomplete outcome data were judged to have a high risk in the study conducted by Jiang et al. (2021); selective reporting was rated as having an unclear risk in the study conducted by Firouzi et al. (2015); and other potential sources of bias were rated as having an unclear risk in the studies conducted by Abbasi et al. (2018) and Miraghajani et al. (2017). All other domains were assessed as low risk.

### 3.4 Meta-analysis

#### 3.4.1 Renal function outcomes

##### 3.4.1.1 SCR

**Figure 4:**
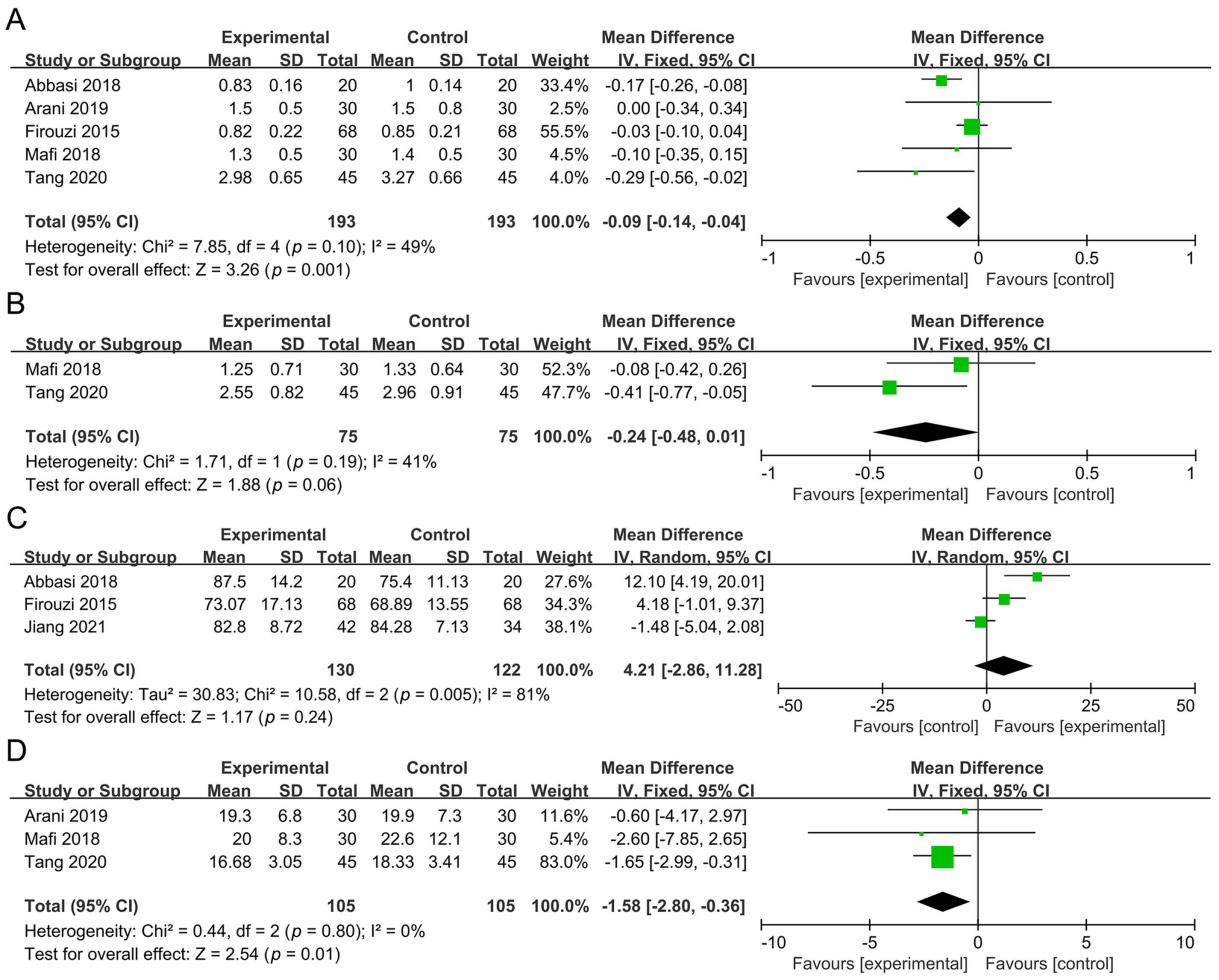
Forest plots of the meta-analysis on renal function outcomes: **(A)** SCR; **(B)** 24 h UP; **(C)** eGFR; **(D)** BUN. SCR, serum creatinine; 24 h UP, 24-h urine protein; eGFR, estimated glomerular filtration rate; BUN, blood urea nitrogen.

Five studies involving 386 participants were included in the meta-analysis of SCR. Compared with the control group, the probiotic group showed a significant reduction in SCR (MD −0.09 mg/dL, 95% CI −0.14 to −0.04, *p* = 0.001, *I*^2^ = 49%) (Figure 4A).

##### 3.4.1.2 24 h UP

Two studies with a total of 150 participants were analyzed for 24 h UP. The results demonstrated no significant difference between the probiotic and control groups (MD −0.24 g, 95% CI −0.48 to 0.01, *p* = 0.06, *I*^2^ = 41%) (Figure 4B).

##### 3.4.1.3 eGFR

Three studies involving 252 participants evaluated eGFR. No significant difference was observed between the probiotic and control groups [MD 4.21 mL/(min*1.73 m^2^), 95% CI −2.86 to 11.28, *p* = 0.24, *I*^2^ = 81%] (Figure 4C).

##### 3.4.1.4 BUN

Three studies with a total of 210 participants were included in the analysis of BUN. Compared with the control group, the probiotic group exhibited a significant reduction in BUN (MD −1.58 mg/dL, 95% CI −2.80 to −0.36, *p* = 0.01, *I*^2^ = 0%) (Figure 4D).

#### 3.4.2 Glucose metabolism outcomes

##### 3.4.2.1 FBG

**Figure 5:**
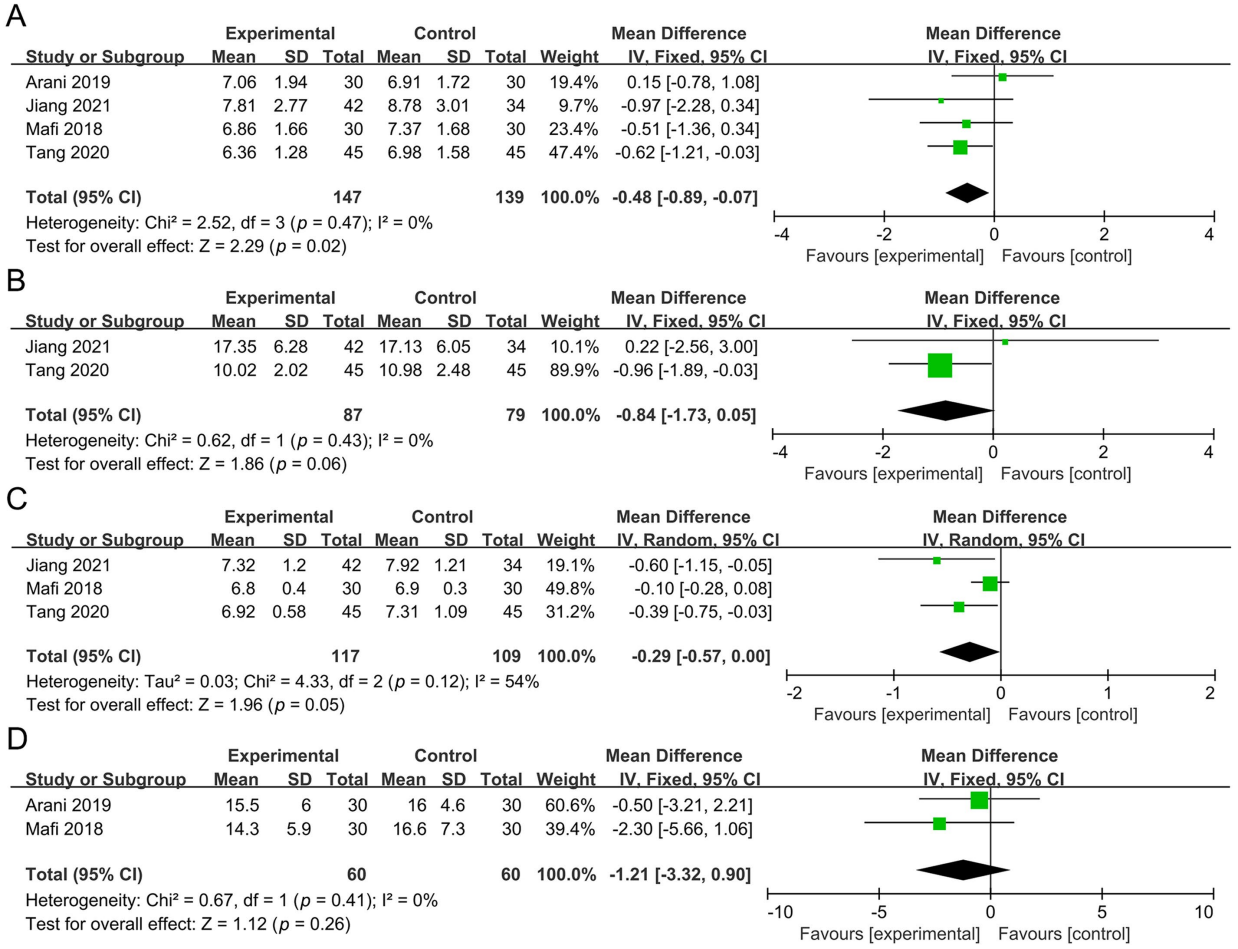
Forest plots of the meta-analysis on glucose metabolism outcomes: **(A)** FBG; **(B)** 2 h PBG; **(C)** HbA1c; **(D)** insulin. FBG, fasting blood glucose; 2 h PBG, 2-h postprandial blood glucose; HbA1c, hemoglobin A1c.

Four studies involving 286 participants were included in the meta-analysis of FBG. Compared with the control group, the probiotic group showed a significant reduction in FBG (MD −0.48 mmol/L, 95% CI −0.89 to −0.07, *p* = 0.02, *I*^2^ = 0%) (Figure 5A).

##### 3.4.2.2 2 h PBG

Two studies with a total of 166 participants were analyzed for 2 h PBG. The results demonstrated no significant difference between the probiotic and control groups (MD −0.84 mmol/L, 95% CI −1.73 to 0.05, *p* = 0.06, *I*^2^ = 0%) (Figure 5B).

##### 3.4.2.3 HbA1c

Two studies with a total of 226 participants were analyzed for HbA1c. The results demonstrated no significant difference between the probiotic and control groups (MD −0.29, 95% CI −0.57 to 0.00, *p* = 0.05, *I*^2^ = 54%) (Figure 5C).

##### 3.4.2.4 Insulin

Two studies with a total of 120 participants were analyzed for insulin. The results demonstrated no significant difference between the probiotic and control groups (MD −1.21 μIU/mL, 95% CI −3.32 to 0.90, *p* = 0.26, *I*^2^ = 0%) (Figure 5D).

#### 3.4.3 Lipid metabolism outcomes

##### 3.4.3.1 TG

**Figure 6:**
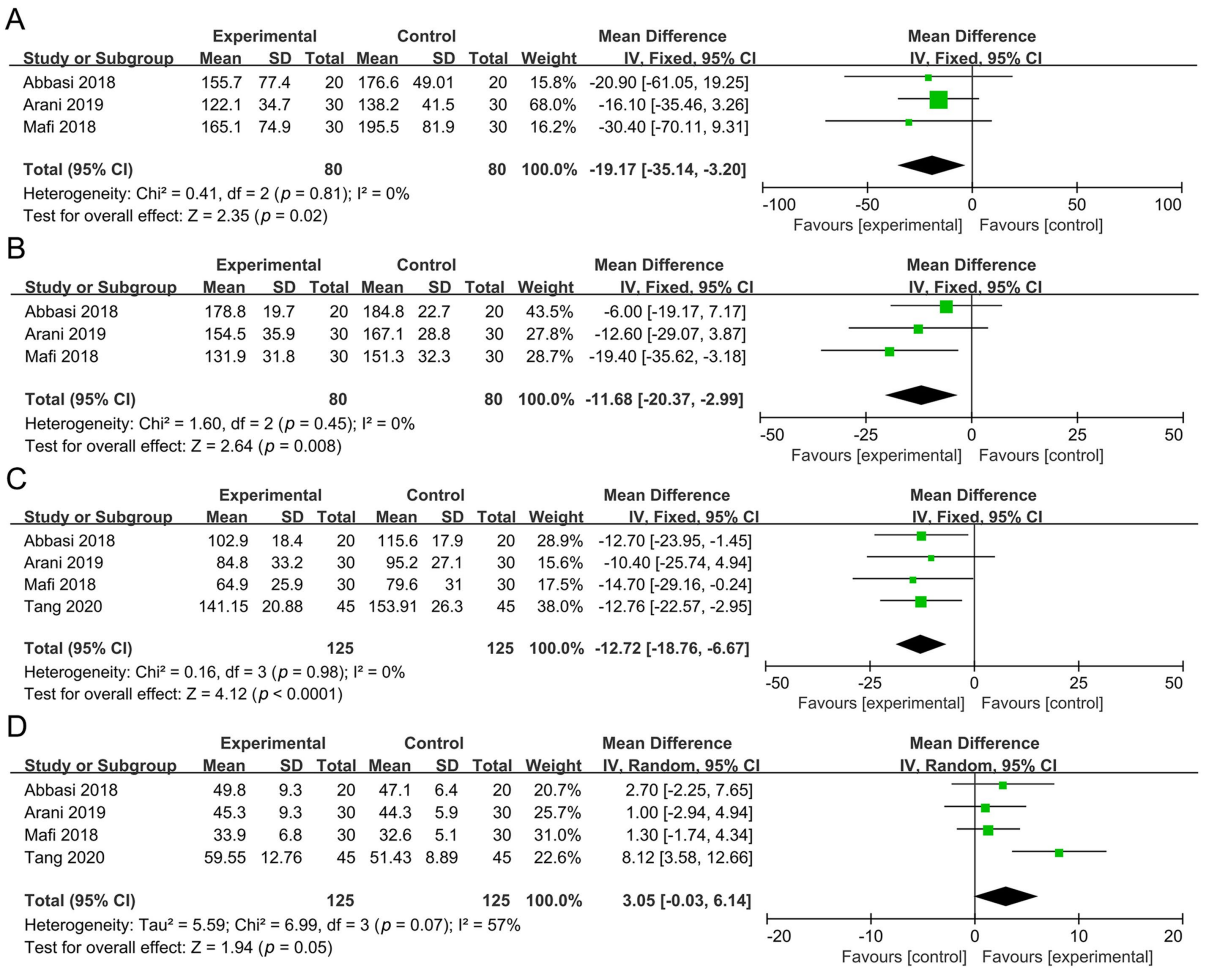
Forest plots of the meta-analysis on lipid metabolism outcomes: **(A)** TG; **(B)** TC; **(C)** LDL-C; **(D)** HDL-C. TG, triglycerides; TC, total cholesterol; LDL-C, low-density lipoprotein cholesterol; HDL-C, high-density lipoprotein cholesterol.

Three studies involving 160 participants were included in the meta-analysis of TG. Compared with the control group, the probiotic group showed a significant reduction in TG (MD −19.17 mg/dL, 95% CI −35.14 to −3.20, *p* = 0.02, *I*^2^ = 0%) (Figure 6A).

##### 3.4.3.2 TC

Three studies involving 160 participants were included in the meta-analysis of TC. Compared with the control group, the probiotic group showed a significant reduction in TC (MD −11.68 mg/dL, 95% CI −20.37 to −2.99, *p* = 0.008, *I*^2^ = 0%) (Figure 6B).

##### 3.4.3.3 LDL-C

Four studies involving 250 participants were included in the meta-analysis of LDL-C. Compared with the control group, the probiotic group showed a significant reduction in LDL-C (MD −12.72 mg/dL, 95% CI −18.76 to −6.67, *p* < 0.0001, *I*^2^ = 0%) (Figure 6C).

##### 3.4.3.4 HDL-C

Four studies involving 250 participants were included in the meta-analysis of HDL-C. No significant difference was observed between the probiotic and control groups (MD 3.05 mg/dL, 95% CI −0.03 to 6.14, *p* = 0.05, *I*^2^ = 57%) (Figure 6D).

#### 3.4.4 Inflammation and oxidative outcomes

##### 3.4.4.1 Hs-CRP

**Figure 7:**
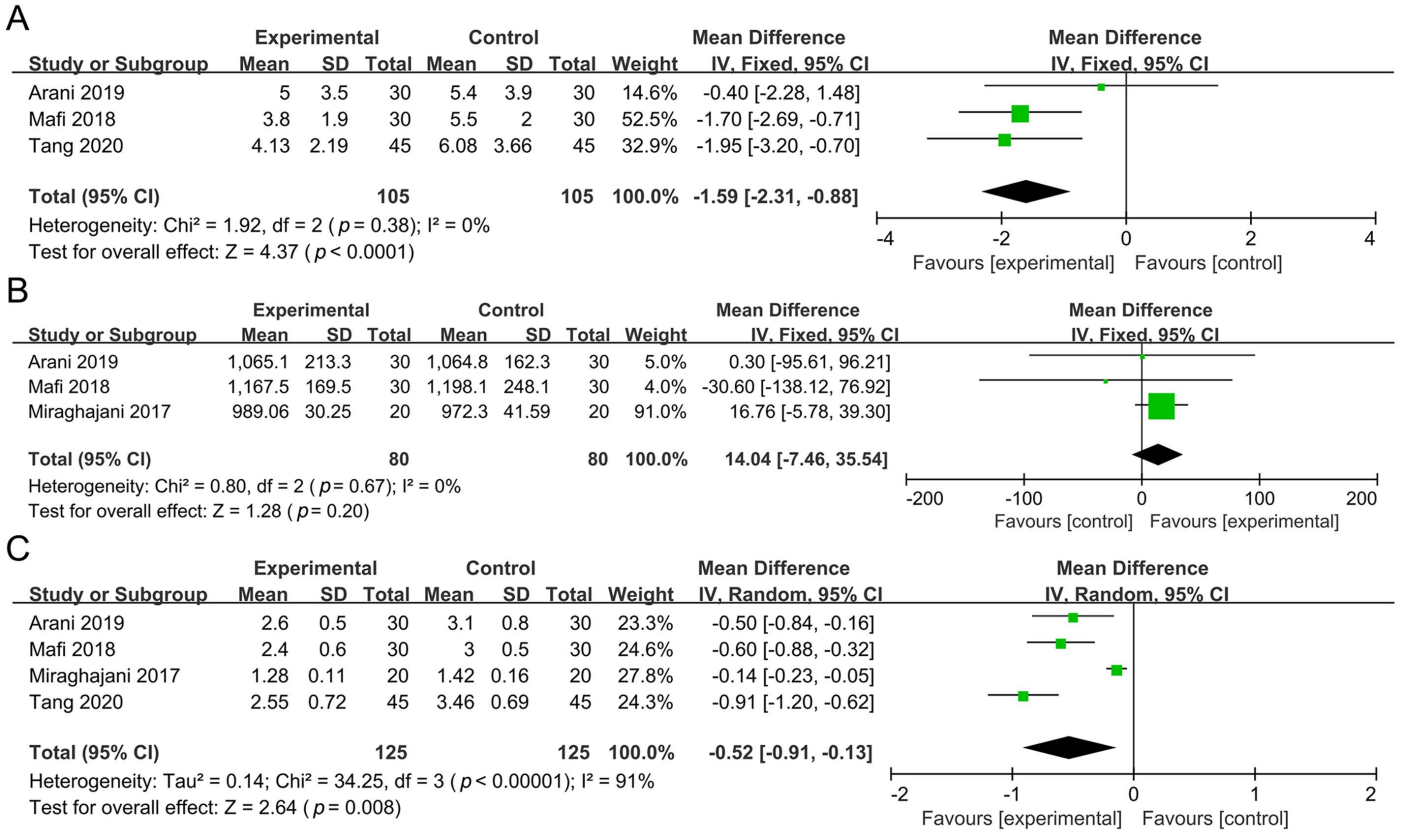
Forest plots of the meta-analysis on inflammation and oxidative outcomes: **(A)** Hs-CRP; **(B)** TAC; **(C)** MDA. Hs-CRP, high-sensitivity C-reactive protein; TAC, total antioxidant capacity; MDA, malondialdehyde.

Three studies involving 210 participants were included in the meta-analysis of hs-CRP. Compared with the control group, the probiotic group showed a significant reduction in hs-CRP (MD −1.59 mg/L, 95% CI −2.31 to −0.88, *p* < 0.0001, *I*^2^ = 0%) (Figure 7A).

##### 3.4.4.2 TAC

Three studies involving 160 participants were included in the meta-analysis of TAC. No significant difference was found between the probiotic and control groups (MD 14.04 mmol/L, 95% CI −7.46 to 35.54, *p* = 0.20, *I*^2^ = 0%) (Figure 7B).

##### 3.4.4.3 MDA

Four studies involving 250 participants were included in the meta-analysis of MDA. Compared with the control group, the probiotic group showed a significant reduction in MDA (MD −0.52 μmol/L, 95% CI −0.91 to −0.13, *p* = 0.008, *I*^2^ = 91%) (Figure 7C).

### 3.5 Sensitivity analysis

High heterogeneity was observed in eGFR, HbA1c, HDL-C, and MDA, and therefore sensitivity analyses were conducted to explore the potential sources of heterogeneity (Table 2). The results indicated that the heterogeneity in HbA1c was attributable to the study by Mafi et al. (2018), which may be explained by differences in participant ethnicity. After excluding this study, the heterogeneity of HbA1c was substantially reduced and the statistical significance of the effect changed (MD −0.45, 95% CI −0.75 to −0.15, *p* = 0.003, *I*^2^ = 0%), suggesting that the HbA1c result was not robust. The heterogeneity in HDL-C was mainly driven by the study of Tang et al. (2020), possibly due to the lack of participant blinding. After excluding this study, the heterogeneity of HDL-C was markedly reduced while the statistical significance remained unchanged (MD 1.48, 95% CI −0.69 to 3.64, *p* = 0.18, *I*^2^ = 0%), indicating that the HDL-C result was robust. The heterogeneity in MDA was attributable to the study of Miraghajani et al. (2017), which may be explained by the relatively short intervention duration. After excluding this study, the heterogeneity of MDA was significantly reduced while the statistical significance was preserved (MD −0.68, 95% CI −0.86 to −0.51, *p* < 0.00001, *I*^2^ = 47%), indicating that the MDA result was robust. Although the sensitivity analysis did not identify the source of heterogeneity for eGFR, it confirmed the robustness of the pooled result. However, since fewer than five studies were included for each of these outcomes, subgroup analyses were not performed.

**Table 2:** Leave-one-out sensitivity analyses of identified heterogeneous sources.

Outcome	Heterogeneity source	Sensitivity analysis results			Robustness
		*I*^2^/%	MD (95% CI)	*p* value	
eGFR	None	—	—	—	Robust
HbA1c	Mafi et al. (2018)	0	−0.45 (−0.75, −0.15)	0.003	Not robust
HDL-C	Tang et al. (2020)	0	1.48 (−0.69, 3.64)	0.18	Robust
MDA	Miraghajani et al. (2017)	47	−0.68 (−0.86, −0.51)	<0.00001	Robust

MD, mean difference; CI, confidence interval; eGFR, estimated glomerular filtration rate; HbA1c, hemoglobin A1c; HDL-C, high-density lipoprotein cholesterol; MDA, malondialdehyde.

### 3.6 Publication bias

**Figure 8:**
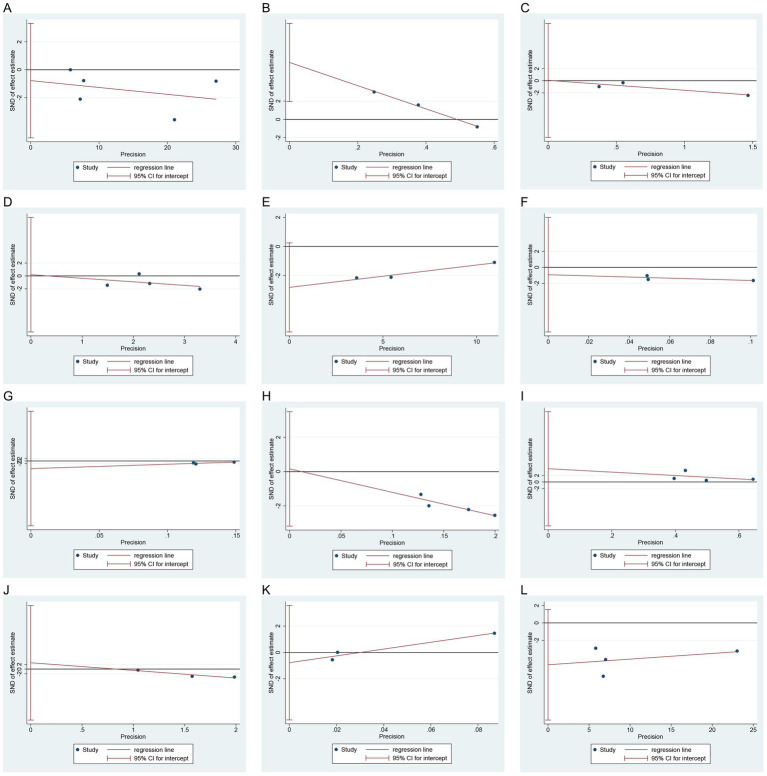
Egger’s test of publication bias: **(A)** SCR; **(B)** eGFR; **(C)** BUN; **(D)** FBG; **(E)** HbA1c; **(F)** TG; **(G)** TC; **(H)** LDL-C; **(I)** HDL-C; **(J)** hs-CRP; **(K)** TAC; **(L)** MDA. SCR, serum creatinine; eGFR, estimated glomerular filtration rate; BUN, blood urea nitrogen; FBG, fasting blood glucose; HbA1c, hemoglobin A1c; TG, triglycerides; TC, total cholesterol; LDL-C, low-density lipoprotein cholesterol; HDL-C, high-density lipoprotein cholesterol; hs-CRP, high-sensitivity C-reactive protein; TAC, total antioxidant capacity; MDA, malondialdehyde.

Egger’s test indicated no significant publication bias for SCR (*p* = 0.589), BUN (*p* = 0.944), FBG (*p* = 0.937), HbA1c (*p* = 0.054), TG (*p* = 0.352), TC (*p* = 0.340), LDL-C (*p* = 0.858), HDL-C (*p* = 0.424), hs-CRP, (*p* = 0.396) TAC (*p* = 0.263), and MDA (*p* = 0.083), while potential publication bias was detected for eGFR (*p* = 0.034) (Figure 8). Since only two studies were included for 24 h UP, 2 h PBG, and insulin, Egger’s test was not performed for these outcomes.

### 3.7 Certainty of evidence

According to the GRADE approach, the certainty of evidence was rated as moderate for SCR, BUN, FBG, TG, TC, LDL-C, hs-CRP, and TAC; low for 24 h UP, 2 h PBG, HbA1c, insulin, HDL-C, and MDA; and very low for eGFR (Table 3). The strength of recommendation was strong.

**Table 3:** Certainty of evidence.

Outcome	Risk of bias	Inconsistency	Indirectness	Imprecision	Publication bias	MD (95% CI)	Certainty of evidence
SCR	None	None	None	Serious	None	−0.09 (−0.14, −0.04)	Moderate
24 h UP	None	None	None	Serious	Suspected	−0.24 (−0.48, 0.01)	Low
eGFR	None	Serious	None	Serious	Suspected	4.21 (−2.86, 11.28)	Very low
BUN	None	None	None	Serious	None	−1.58 (−2.80, −0.36)	Moderate
FBG	None	None	None	Serious	None	−0.48 (−0.89, −0.07)	Moderate
2 h PBG	None	None	None	Serious	Suspected	−0.84 (−1.73, 0.05)	Low
HbA1c	None	Serious	None	Serious	None	−0.29 (−0.57, 0.00)	Low
Insulin	None	None	None	Serious	Suspected	−1.21 (−3.32, 0.90)	Low
TG	None	None	None	Serious	None	−19.17 (−35.14, −3.20)	Moderate
TC	None	None	None	Serious	None	−11.68 (−20.37, −2.99)	Moderate
LDL-C	None	None	None	Serious	None	−12.72 (−18.76, −6.67)	Moderate
HDL-C	None	Serious	None	Serious	None	3.05 (−0.03, 6.14)	Low
hs-CRP	None	None	None	Serious	None	−1.59 (−2.31, −0.88)	Moderate
TAC	None	None	None	Serious	None	14.04 (−7.46, 35.54)	Moderate
MDA	None	Serious	None	Serious	None	−0.52 (−0.91, −0.13)	Low

MD, mean difference; CI, confidence interval; SCR, serum creatinine; 24 h UP, 24-h urine protein; eGFR, estimated glomerular filtration rate; BUN, blood urea nitrogen; FBG, fasting blood glucose; 2 h PBG, 2-h postprandial blood glucose; HbA1c, hemoglobin A1c; TG, triglycerides; TC, total cholesterol; LDL-C, low-density lipoprotein cholesterol; HDL-C, high-density lipoprotein cholesterol; hs-CRP, high-sensitivity C-reactive protein; TAC, total antioxidant capacity; MDA, malondialdehyde.

## 4 Discussion

### 4.1 Research significance and findings

DKD is the most severe microvascular complication of diabetes and one of the leading causes of death among diabetic patients (Thomas et al., 2015). With the discovery of the “gut–kidney axis” (Jiang et al., 2025), probiotics have been regarded as a promising complementary therapy for DKD (Zhang et al., 2025; Wang et al., 2025). However, previous clinical trials and meta-analyses have reported inconsistent findings regarding renal and glycemic outcomes, highlighting the controversy in this field. Tarrahi et al. (2022) reported that probiotics significantly reduced FBG and SCR levels in patients with DKD but had no significant effects on HbA1c or BUN. In contrast, Dai et al. (2022) found that probiotics improved multiple renal and glycemic parameters, including reductions in FBG, HbA1c, SCR, and BUN. Notably, both Tarrahi et al. (2022) and Dai et al. (2022) included a clinical trial conducted in uremic patients, which may have introduced additional confounding factors. Furthermore, Tarrahi et al. (2022) only included studies published up to 2019, resulting in temporal limitations.

Therefore, we conducted an updated meta-analysis to more comprehensively and accurately evaluate the effects of probiotics in patients with DKD. Our results demonstrated that probiotic supplementation significantly reduced SCR, BUN, FBG, TG, TC, LDL-C, hs-CRP, and MDA levels in patients with DKD. Notably, no publication bias was detected for these positive outcomes, and except for MDA, the certainty of evidence was rated as moderate, suggesting that these findings are robust and reliable.

### 4.2 Effect of probiotics on renal function and glycolipid metabolism

Renal function is a key indicator for assessing the progression and prognosis of DKD. The results of this meta-analysis showed that, compared with placebo, probiotic supplementation significantly reduced SCR and BUN levels in patients with DKD, while no significant effects were observed on 24 h UP or eGFR. In previous meta-analyses, Dai et al. (2022) found that probiotics decreased SCR (MD 0.17, 95% CI 0.29 to 0.05) and BUN levels (MD 1.36, 95% CI 2.20 to 0.52) in DKD patients, supporting our findings. However, Tarrahi et al. (2022) reported that probiotics only reduced SCR levels (MD −0.18, 95% CI −0.26 to −0.09), with no significant effect on BUN. AbdelQadir et al. (2020) found no significant effect of probiotics on either SCR or eGFR, presenting conflicting results. These discrepancies may be attributed to differences in participant characteristics and sample sizes. For one, both Tarrahi et al. (2022) and AbdelQadir et al. (2020) included the study by Soleimani et al. (2017), which recruited uremic DKD patients, potentially introducing additional confounding factors that affected the pooled results. In contrast, we excluded the study by Soleimani et al. (2017), thereby reducing the confounding effects of severe renal impairment and dialysis-related metabolic disturbances. For another, Tarrahi et al. (2022) and AbdelQadir et al. (2020) included only four studies when analyzing SCR, which may have limited the statistical power of their analyses. In comparison, our meta-analysis included five RCTs involving 386 participants, enhancing the robustness of the findings. Collectively, these results suggest that probiotics have the potential to reduce SCR and BUN levels in patients with DKD, though their effects on 24 h UP and eGFR remain limited.

Abnormal glucose metabolism is a central driver of the onset and progression of DKD (Stanton, 2021). Our meta-analysis demonstrated that probiotics significantly reduced FBG levels, while no significant effects were observed on 2 h PBG, HbA1c, or insulin levels. Sensitivity analysis revealed that the results for HbA1c were unstable, with heterogeneity largely influenced by the study of Mafi et al. (2018). After excluding this study, HbA1c showed a significant between-group difference (MD −0.45, 95% CI −0.75 to −0.15). We speculate that this variation may be associated with ethnic differences. Among the three included studies reporting HbA1c, only Mafi et al. (2018) recruited Iranian participants, whereas the other two studies (Jiang et al., 2021; Tang et al., 2020) were conducted in Chinese populations. In previous meta-analyses, Tarrahi et al. (2022) found that probiotics significantly reduced FPG levels (MD −19.08, 95% CI −32.16 to −5.99) but had no significant effects on HbA1c or insulin. Similarly, AbdelQadir et al. (2020) also reported no significant impact of probiotics on serum insulin levels (MD −1.99, 95% CI −3.99 to 0.01), supporting our findings. Interestingly, Dai et al. (2022) observed that probiotics not only reduced FBG levels but also significantly lowered HbA1c levels in DKD patients. The inconsistent results regarding HbA1c may stem from additional confounding factors. Unlike Dai et al. (2022), our analysis excluded the study by Soleimani et al. (2017) when pooling HbA1c data, which altered the statistical significance. Given that Soleimani et al. (2017) focused on uremic DKD patients, including this study may have introduced confounding bias and led to a false-positive effect. Overall, current evidence suggests that probiotics effectively reduce FBG levels in patients with DKD, while their effects on 2 h PBG, HbA1c, and insulin levels warrant further investigation.

Lipid metabolism disorders are key pathogenic mechanisms in DKD (Han et al., 2024). Our meta-analysis demonstrated that probiotics significantly reduced TG, TC, and LDL-C levels in DKD patients, while showing no significant effect on HDL-C levels. Sensitivity analysis revealed that the heterogeneity of HDL-C was mainly influenced by the study of Tang et al. (2020). After excluding this study, heterogeneity markedly decreased without altering statistical significance (MD 1.48, 95% CI −0.69 to 3.64), suggesting that the result was robust. However, previous meta-analyses have reported conflicting findings. Dai et al. (2022) found that probiotics significantly decreased TC (MD −6.93, 95% CI −11.67 to −2.19) and LDL-C levels (MD −7.14, 95% CI −11.03 to −3.24) and increased HDL-C levels (MD 2.72, 95% CI 0.47 to 4.97) in DKD patients. In contrast, AbdelQadir et al. (2020) reported that probiotics only reduced LDL-C (MD −6.60, 95% CI −13.11 to −0.10), without significant improvements in TC, TG, or HDL-C. Notably, compared with Dai et al. (2022), our exclusion of the study by Soleimani et al. (2017) yielded a nonsignificant result for HDL-C, suggesting that the previously reported benefit in HDL-C might have been confounded and unstable. Additionally, compared with AbdelQadir et al. (2020), our inclusion of additional clinical trials by Abbasi et al. (2018) and Tang et al. (2020) clarified the beneficial effects of probiotics on TG and TC levels, indicating that the previously negative results may have been due to limited sample sizes. Collectively, the current evidence supports the potential of probiotics to reduce TG, TC, and LDL-C levels in DKD patients, while their effects on HDL-C require further investigation.

The mechanisms underlying the improvement of renal function and glucose–lipid metabolism by probiotics in DKD patients are mainly associated with modulation of gut microbiota composition and its metabolites. Dysbiosis in DKD is characterized by a reduction in beneficial short-chain fatty acid (SCFA)-producing bacteria (Sabatino et al., 2017). Compared with healthy individuals, DKD patients exhibit increased abundance of *Proteobacteria* and *Fusobacteria* at the phylum level, and *Escherichia-Shigella*, *Desulfovibrio*, and *Streptococcus* at the genus level, whereas the abundance of *Roseburia*, *Faecalibacterium*, *Pyramidobacter*, *Prevotellaceae*_UCG-001, and *Prevotella*_9 is reduced (Zhao et al., 2021). *Roseburia* spp., *Faecalibacterium prausnitzii*, and *Prevotella* are known SCFA producers (Jiang et al., 2017). SCFAs can stimulate glucagon-like peptide-1 secretion and protect against chronic hyperglycemia induced by renal oxidative stress (Everard and Cani, 2014). Further evidence suggests that gut microbiota-derived acetate can modulate glucose metabolism, reduce glycotoxin accumulation, and improve mitochondrial function, thereby alleviating glomerular injury and renal fibrosis (Luo et al., 2022). Additionally, metabolomic studies have revealed that plasma bile acid levels in DKD patients increase progressively and are closely correlated with disease progression (Zhang et al., 2024). Gut microbiota such as Lactobacillus, Clostridium, Bifidobacterium, and Enterococcus can regulate bile acid metabolism and cholesterol secretion, thereby attenuating renal inflammation (Tian et al., 2020).

### 4.3 Effect of probiotics on inflammation and oxidative stress

In addition to disturbances in glucose–lipid metabolism, inflammation and oxidative stress are key drivers of renal injury in DKD (Su et al., 2023; Rana et al., 2022). Our meta-analysis revealed that while probiotics did not significantly affect TAC levels in DKD patients, they significantly reduced hs-CRP and MDA levels, consistent with the findings of Dai et al. (2022). Sensitivity analysis indicated that the high heterogeneity of MDA was mainly associated with the study by Miraghajani et al. (2017) After excluding this study, the heterogeneity of MDA markedly decreased, while statistical significance remained unchanged (MD −0.68, 95% CI −0.86 to −0.51), suggesting that this result was robust. Notably, AbdelQadir et al. (2020) and Bohlouli et al. (2021) both reported that probiotics not only significantly decreased hs-CRP and MDA levels but also increased TAC levels. The discrepancy in TAC results may be attributed to heterogeneity among participants. Specifically, both AbdelQadir et al. (2020) and Bohlouli et al. (2021) included the study by Soleimani et al. (2017), which was conducted in uremic patients and may have exaggerated the difference in TAC between the probiotic and placebo groups. Therefore, although probiotics were found to significantly reduce hs-CRP and MDA levels, their effects on TAC warrant further investigation through large-scale RCTs.

These findings reflect the anti-inflammatory and antioxidant properties of probiotics, which may be mediated by restoring intestinal barrier integrity and preventing lipopolysaccharide (LPS) translocation. On the one hand, gut dysbiosis disrupts intestinal barrier function, promoting the accumulation of inflammatory mediators and uremic toxins such as indoxyl sulfate and trimethylamine N-oxide (Wang et al., 2025; Sun et al., 2022). On the other hand, dysbiosis-induced LPS translocation triggers systemic inflammation, oxidative stress, and immune cell infiltration, leading to renal inflammation and fibrosis (Jiang et al., 2025). Conversely, probiotics may restore intestinal barrier integrity and suppress LPS translocation, thereby mitigating renal inflammation, oxidative stress, and fibrosis to exert renoprotective effects (Wang et al., 2016).

### 4.4 Clinical discovery and inspiration

Compared with previous meta-analyses, our study offers several novel findings and methodological improvements. First, unlike earlier analyses by AbdelQadir et al. (2020), Bohlouli et al. (2021), and Tarrahi et al. (2022), our meta-analysis included a broader and more up-to-date range of RCTs, providing a more comprehensive and precise assessment of probiotic efficacy in DKD. Importantly, compared with the meta-analyses by Tarrahi et al. (2022) and Dai et al. (2022), we excluded studies involving uremic patients, such as that by Soleimani et al. (2017), to minimize potential confounding caused by severe renal impairment and dialysis-related metabolic disturbances. This refinement allowed us to more accurately evaluate the true effect of probiotics on DKD rather than on end-stage renal disease. Consequently, our findings revealed that probiotics significantly improved renal function (reducing SCR and BUN levels), glycemic control (lowering FBG), and lipid metabolism (reducing TG, TC, and LDL-C levels), while also exerting anti-inflammatory and antioxidant effects (decreasing hs-CRP and MDA). Moreover, the certainty of evidence for most positive outcomes was moderate and free from publication bias, which enhances the reliability of these conclusions.

From a clinical perspective, this meta-analysis provides several valuable insights. The results support probiotics as a promising complementary therapy for patients with DKD, supplementing standard hypoglycemic and renoprotective treatments. By improving renal biochemical markers, glycemic and lipid metabolism, and attenuating inflammation and oxidative stress, probiotics may help delay DKD progression and improve overall metabolic homeostasis. These findings suggest that probiotics could be particularly beneficial for patients with early or moderate DKD, who may achieve renal and metabolic protection through modulation of the gut–kidney axis. Clinicians may therefore consider probiotic supplementation, particularly strains with proven efficacy in restoring short-chain fatty acid-producing bacteria and enhancing intestinal barrier function, as part of a personalized and multi-target therapeutic strategy. Future large-scale, strain-specific RCTs are warranted to determine the optimal probiotic formulations, dosages, and treatment durations, and to clarify the underlying mechanisms linking gut microbiota modulation with renal outcomes in DKD.

### 4.5 Limitations and prospects

Despite the robustness of our findings, several limitations should be acknowledged. First, all included studies were conducted in Iran, Malaysia, and China, which may limit the external applicability of the results to other populations with different genetic backgrounds, dietary patterns, and lifestyles. Second, although our meta-analysis incorporated the most recent and comprehensive collection of RCTs to date, the total sample size of DKD patients remains relatively small, which may reduce the statistical power and generalizability of the conclusions. Third, heterogeneity was observed in several outcomes, which could be attributed to variations in probiotic strains, dosages, intervention durations, disease stages, and baseline characteristics of participants. Fourth, most included studies did not provide detailed information on dietary habits, medication use, or gut microbiota composition, making it difficult to assess potential confounding factors or to identify the specific microbial alterations responsible for clinical benefits. Fifth, the intervention durations in most trials were relatively short, which may not fully capture the long-term renal and metabolic effects of probiotics. Additionally, publication bias cannot be completely excluded, as studies with negative or null findings are less likely to be published.

Future research should aim to conduct large-scale, high-quality, multicenter RCTs across diverse populations with standardized probiotic strains, dosages, and intervention durations to validate and extend these findings. Further investigations are warranted to explore strain-specific effects and potential synergistic combinations that maximize renal protection in DKD. The integration of metagenomic, metabolomic, and transcriptomic analyses could help elucidate the mechanistic pathways linking gut microbiota modulation to renal and metabolic outcomes. Moreover, future studies should assess the long-term efficacy and safety of probiotics across different stages of DKD and in conjunction with conventional therapies. Such efforts will provide stronger evidence to support the clinical application of probiotics as a safe, accessible, and mechanism-based complementary therapy for DKD.

## 5 Conclusion

Probiotic supplementation may benefit renal function, glycemic control, lipid metabolism, and inflammation/oxidative stress in patients with DKD. Given their multi-faceted effects, probiotics represent a promising adjunctive therapy for DKD. Nevertheless, further large-scale, high-quality randomized controlled trials are needed to validate these findings.

## Data Availability

The original contributions presented in the study are included in the article/supplementary material, further inquiries can be directed to the corresponding author.

## References

[ref1] Abbasi B. Mirlohi M. Daniali M. Ghiasvand R. (2018). Effects of probiotic soy milk on lipid panel in type 2 diabetic patients with nephropathy: a double-blind randomized clinical trial. Prog. Nutr. 20, 70–78. doi: 10.23751/pn.v20i2-S.5342

[ref2] AbdelQadir Y. H. Hamdallah A. Sibaey E. A. Hussein A. S. Abdelaziz M. AbdelAzim A. . (2020). Efficacy of probiotic supplementation in patients with diabetic nephropathy: a systematic review and meta-analysis. Clin. Nutr. ESPEN 40, 57–67. doi: 10.1016/j.clnesp.2020.06.019, 33183573

[ref3] Agarwal R. Green J. B. Heerspink H. J. L. Mann J. F. E. McGill J. B. Mottl A. K. . (2025). Impact of baseline GLP-1 receptor agonist use on albuminuria reduction and safety with simultaneous initiation of finerenone and empagliflozin in type 2 diabetes and chronic kidney disease (CONFIDENCE trial). Diabetes Care 48, 1904–1913. doi: 10.2337/dc25-1673, 40968755 PMC12583382

[ref4] Aguilera-Martínez S. L. Muñoz-Ortega M. H. Martínez-Hernández S. L. Morones-Gamboa J. C. Ventura-Juárez J. (2025). Modulation of mesangial cells by tamsulosin and pioglitazone under hyperglycemic conditions: an *in vitro* and *in vivo* study. Int. J. Mol. Sci. 26:9277. doi: 10.3390/ijms26199277, 41096550 PMC12524318

[ref5] Apte M. Zambre S. Pisar P. Roy B. Tupe R. (2024). Decoding the role of aldosterone in glycation-induced diabetic complications. Biochem. Biophys. Res. Commun. 721:150107. doi: 10.1016/j.bbrc.2024.150107, 38781658

[ref6] Bhagwat Y. Kumar S. (2023). A review on case burden of diabetes mellitus before and after the implementation of national programme for prevention and control of cancer, diabetes, cardiovascular diseases and stroke. Cureus 15:e49446. doi: 10.7759/cureus.49446, 38149165 PMC10751034

[ref7] Bohlouli J. Namjoo I. Borzoo-Isfahani M. Hojjati Kermani M. A. Balouch Zehi Z. Moravejolahkami A. R. (2021). Effect of probiotics on oxidative stress and inflammatory status in diabetic nephropathy: a systematic review and meta-analysis of clinical trials. Heliyon 7:e05925. doi: 10.1016/j.heliyon.2021.e05925, 33490683 PMC7808957

[ref8] Cai L. Huang Y. Li X. Cao D. Liu F. (2024). Effects of dietary intervention on diabetic nephropathy: an umbrella review of systematic reviews and meta-analyses of randomized controlled trials. Front. Endocrinol. 15:1385872. doi: 10.3389/fendo.2024.1385872, 38742202 PMC11089238

[ref9] Dai Y. Quan J. Xiong L. Luo Y. Yi B. (2022). Probiotics improve renal function, glucose, lipids, inflammation and oxidative stress in diabetic kidney disease: a systematic review and meta-analysis. Ren. Fail. 44, 862–880. doi: 10.1080/0886022X.2022.2079522, 35611435 PMC9154786

[ref10] de Boer I. H. Khunti K. Sadusky T. Tuttle K. R. Neumiller J. J. Rhee C. M. . (2022). Diabetes management in chronic kidney disease: a consensus report by the American Diabetes Association (ADA) and kidney disease: improving global outcomes (KDIGO). Diabetes Care 45, 3075–3090. doi: 10.2337/dci22-0027, 36189689 PMC9870667

[ref11] Di Marco M. Scilletta S. Miano N. Marrano N. Natalicchio A. Giorgino F. . (2023). Cardiovascular risk and renal injury profile in subjects with type 2 diabetes and non-albuminuric diabetic kidney disease. Cardiovasc. Diabetol. 22:344. doi: 10.1186/s12933-023-02065-2, 38093293 PMC10720121

[ref12] Dwivedi S. Sikarwar M. S. (2025). Diabetic nephropathy: pathogenesis, mechanisms, and therapeutic strategies. Horm. Metab. Res. 57, 7–17. doi: 10.1055/a-2435-8264, 39572154

[ref13] Elian V. Popovici V. Nicolescu M. I. Nicolescu A. M. Aurelian S. M. Ozon E. A. (2025). Interconnected mechanistic pathways, molecular biomarkers, and therapeutic approach of oral cancer in patients with diabetes mellitus. Curr. Issues Mol. Biol. 47:929. doi: 10.3390/cimb47110929, 41296433 PMC12651534

[ref14] Everard A. Cani P. D. (2014). Gut microbiota and GLP-1. Rev. Endocr. Metab. Disord. 15, 189–196. doi: 10.1007/s11154-014-9288-6, 24789701

[ref15] Firouzi S. Mohd-Yusof B.-N. Majid H.-A. Ismail A. Kamaruddin N.-A. (2015). Effect of microbial cell preparation on renal profile and liver function among type 2 diabetics: a randomized controlled trial. BMC Complement. Altern. Med. 15:433. doi: 10.1186/s12906-015-0952-5, 26654906 PMC4676823

[ref16] Gaddy A. Elrggal M. Madariaga H. Kelly A. Lerma E. Colbert G. B. (2025). Diabetic kidney disease. Dis. Mon. 71:101848. doi: 10.1016/j.disamonth.2024.101848, 39753456

[ref17] Gupta S. Dominguez M. Golestaneh L. (2023). Diabetic kidney disease: an update. Med. Clin. North Am. 107, 689–705. doi: 10.1016/j.mcna.2023.03.004, 37258007

[ref18] Han Y.-Z. Du B.-X. Zhu X.-Y. Wang Y.-Z.-Y. Zheng H.-J. Liu W.-J. (2024). Lipid metabolism disorder in diabetic kidney disease. Front. Endocrinol. 15:1336402. doi: 10.3389/fendo.2024.1336402, 38742197 PMC11089115

[ref19] Higgins J. P. T. Altman D. G. Gøtzsche P. C. Jüni P. Moher D. Oxman A. D. . (2011). The Cochrane Collaboration’s tool for assessing risk of bias in randomised trials. BMJ 343:d5928. doi: 10.1136/bmj.d5928, 22008217 PMC3196245

[ref20] Hou G. Dong Y. Jiang Y. Zhao W. Zhou L. Cao S. . (2025). Immune inflammation and metabolic interactions in the pathogenesis of diabetic nephropathy. Front. Endocrinol. 16:1602594. doi: 10.3389/fendo.2025.1602594, 40698245 PMC12279506

[ref21] International Diabetes Federation (2025). IDF diabetes atlas. 11th Edn. Brussels: International Diabetes Federation.

[ref22] Jiang H. Wang X. Zhou W. Huang Z. Zhang W. (2025). Gut microbiota dysbiosis in diabetic nephropathy: mechanisms and therapeutic targeting via the gut-kidney axis. Front. Endocrinol. 16:1661037. doi: 10.3389/fendo.2025.1661037, 41048436 PMC12488459

[ref23] Jiang S. Xie S. Lv D. Wang P. He H. Zhang T. . (2017). Alteration of the gut microbiota in Chinese population with chronic kidney disease. Sci. Rep. 7:2870. doi: 10.1038/s41598-017-02989-2, 28588309 PMC5460291

[ref24] Jiang H. Zhang Y. Xu D. Wang Q. (2021). Probiotics ameliorates glycemic control of patients with diabetic nephropathy: a randomized clinical study. J. Clin. Lab. Anal. 35:e23650. doi: 10.1002/jcla.23650, 33666270 PMC8059722

[ref25] Jin Z.-C. Zhou X.-H. He J. (2015). Statistical methods for dealing with publication bias in meta-analysis. Stat. Med. 34, 343–360. doi: 10.1002/sim.6342, 25363575

[ref26] Joseph J. (2025). Comparative effectiveness of SGLT2 inhibitors and semaglutide in diabetic nephropathy: a retrospective observational study. Cureus 17:e87399. doi: 10.7759/cureus.87399, 40772215 PMC12326033

[ref27] Li J. Liu Y. Chen S. Dai X. Wang J. (2025). Pharmacological agents for procedural sedation and analgesia in patients undergoing gastrointestinal endoscopy: a systematic review and network meta-analysis. EClinicalMedicine 85:103307. doi: 10.1016/j.eclinm.2025.103307, 40599871 PMC12209896

[ref28] Li X.-T. Yun M.-Z. (2025). The impact of sulfonylureas on diverse ion channels: an alternative explanation for the antidiabetic actions. Front. Cell Dev. Biol. 13:1528369. doi: 10.3389/fcell.2025.1528369, 40625684 PMC12231142

[ref29] Lin L. Chu H. (2018). Quantifying publication bias in meta-analysis. Biometrics 74, 785–794. doi: 10.1111/biom.12817, 29141096 PMC5953768

[ref30] Liu Z. Liu J. Wang W. An X. Luo L. Yu D. . (2023). Epigenetic modification in diabetic kidney disease. Front. Endocrinol. 14:1133970. doi: 10.3389/fendo.2023.1133970, 37455912 PMC10348754

[ref31] Liu X. Zhang C. Fu Y. Xie L. Kong Y. Yang X. (2025). Inflammation, apoptosis, and fibrosis in diabetic nephropathy: molecular crosstalk in proximal tubular epithelial cells and therapeutic implications. Curr. Issues Mol. Biol. 47:885. doi: 10.3390/cimb47110885, 41296389 PMC12651756

[ref32] Luo L. Luo J. Cai Y. Fu M. Li W. Shi L. . (2022). Inulin-type fructans change the gut microbiota and prevent the development of diabetic nephropathy. Pharmacol. Res. 183:106367. doi: 10.1016/j.phrs.2022.106367, 35882293

[ref33] Mafi A. Namazi G. Soleimani A. Bahmani F. Aghadavod E. Asemi Z. (2018). Metabolic and genetic response to probiotics supplementation in patients with diabetic nephropathy: a randomized, double-blind, placebo-controlled trial. Food Funct. 9, 4763–4770. doi: 10.1039/c8fo00888d, 30113051

[ref34] Mazruei Arani N. Emam-Djomeh Z. Tavakolipour H. Sharafati-Chaleshtori R. Soleimani A. Asemi Z. (2019). The effects of probiotic honey consumption on metabolic status in patients with diabetic nephropathy: a randomized, double-blind, controlled trial. Probiotics Antimicrob. Proteins 11, 1195–1201. doi: 10.1007/s12602-018-9468-x, 30218286

[ref35] Migliavaca C. B. Stein C. Colpani V. Barker T. H. Ziegelmann P. K. Munn Z. . (2022). Meta-analysis of prevalence: *I*^2^ statistic and how to deal with heterogeneity. Res. Synth. Methods 13, 363–367. doi: 10.1002/jrsm.1547, 35088937

[ref36] Miraghajani M. Zaghian N. Mirlohi M. Feizi A. Ghiasvand R. (2017). The impact of probiotic soy milk consumption on oxidative stress among type 2 diabetic kidney disease patients: a randomized controlled clinical trial. J. Ren. Nutr. 27, 317–324. doi: 10.1053/j.jrn.2017.04.004, 28579313

[ref37] Młynarska E. Buławska D. Czarnik W. Hajdys J. Majchrowicz G. Prusinowski F. . (2024). Novel insights into diabetic kidney disease. Int. J. Mol. Sci. 25:10222. doi: 10.3390/ijms251810222, 39337706 PMC11432709

[ref38] Natale P. Green S. C. Tunnicliffe D. J. Pellegrino G. Toyama T. Sarafidis P. . (2025). Thiazolidinediones for people with chronic kidney disease and diabetes. Cochrane Database Syst. Rev. 2025:CD015907. doi: 10.1002/14651858.CD015907.pub2, 41231410 PMC12614201

[ref39] Page M. J. McKenzie J. E. Bossuyt P. M. Boutron I. Hoffmann T. C. Mulrow C. D. . (2021). The PRISMA 2020 statement: an updated guideline for reporting systematic reviews. BMJ 372:n71. doi: 10.1136/bmj.n71 33782057 PMC8005924

[ref40] Patsopoulos N. A. Evangelou E. Ioannidis J. P. (2008). Sensitivity of between-study heterogeneity in meta-analysis: proposed metrics and empirical evaluation. Int. J. Epidemiol. 37, 1148–1157. doi: 10.1093/ije/dyn065, 18424475 PMC6281381

[ref41] Prasad M. (2024). Introduction to the GRADE tool for rating certainty in evidence and recommendations. Clin. Epidemiol. Glob. Health 25:101484. doi: 10.1016/j.cegh.2023.101484

[ref42] Rana R. Manoharan J. Gupta A. Gupta D. Elwakiel A. Khawaja H. . (2022). Activated protein C ameliorates tubular mitochondrial reactive oxygen species and inflammation in diabetic kidney disease. Nutrients 14:3138. doi: 10.3390/nu14153138, 35956315 PMC9370435

[ref43] Ratan Y. Rajput A. Pareek A. Pareek A. Singh G. (2025). Comprehending the role of metabolic and hemodynamic factors alongside different signaling pathways in the pathogenesis of diabetic nephropathy. Int. J. Mol. Sci. 26:3330. doi: 10.3390/ijms26073330, 40244213 PMC11989741

[ref44] Richardson M. Garner P. Donegan S. (2019). Interpretation of subgroup analyses in systematic reviews: a tutorial. Clin. Epidemiol. Glob. Health 7, 192–198. doi: 10.1016/j.cegh.2018.05.005

[ref45] Sabatino A. Regolisti G. Cosola C. Gesualdo L. Fiaccadori E. (2017). Intestinal microbiota in type 2 diabetes and chronic kidney disease. Curr. Diab. Rep. 17:16. doi: 10.1007/s11892-017-0841-z, 28271466

[ref46] Sanglard A. Miranda B. C. B. Vieira A. L. F. Macedo M. V. M. Santos R. L. Campos A. S. R. R. . (2025). The role of renin-angiotensin system in diabetic nephropathy: an update. Mini Rev. Med. Chem. 25, 591–600. doi: 10.2174/0113895575344980250130062547, 39936412

[ref47] Soleimani A. Zarrati Mojarrad M. Bahmani F. Taghizadeh M. Ramezani M. Tajabadi-Ebrahimi M. . (2017). Probiotic supplementation in diabetic hemodialysis patients has beneficial metabolic effects. Kidney Int. 91, 435–442. doi: 10.1016/j.kint.2016.09.040, 27927601

[ref48] Stanton R. C. (2021). Role of glucose metabolism and mitochondrial function in diabetic kidney disease. Curr. Diab. Rep. 21:6. doi: 10.1007/s11892-020-01372-2, 33449215

[ref49] Su S. Ma Z. Wu H. Xu Z. Yi H. (2023). Oxidative stress as a culprit in diabetic kidney disease. Life Sci. 322:121661. doi: 10.1016/j.lfs.2023.121661, 37028547

[ref50] Sun X. Chen J. Huang Y. Zhu S. Wang S. Xu Z. . (2022). Yishen Qingli Heluo granule ameliorates renal dysfunction in 5/6 nephrectomized rats by targeting gut microbiota and intestinal barrier integrity. Front. Pharmacol. 13:858881. doi: 10.3389/fphar.2022.858881, 35814258 PMC9258868

[ref51] Tang W. Liu N. Fan Y. (2020). Effect of probiotics supplementation on the risk of disease progression in elderly with diabetic nephropathy. Chin. J. Microecol. 32, 570–574. doi: 10.13381/j.cnki.cjm.202005016

[ref52] Tarrahi M. J. Namjoo I. Borzoo-Isfahani M. Ebdali H. Moravejolahkami A. R. (2022). Can probiotics supplementation improve glycemic and renal status in diabetic nephropathy? A systematic review and meta-analysis of clinical trials. Endocr. Metab. Immune Disord. Drug Targets 22, 143–158. doi: 10.2174/1871530321666210121154037, 33475080

[ref53] Thomas M. C. Brownlee M. Susztak K. Sharma K. Jandeleit-Dahm K. A. Zoungas S. . (2015). Diabetic kidney disease. Nat. Rev. Dis. Primers 1:15018. doi: 10.1038/nrdp.2015.18 27188921 PMC7724636

[ref54] Tian Y. Gui W. Koo I. Smith P. B. Allman E. L. Nichols R. G. . (2020). The microbiome modulating activity of bile acids. Gut Microbes 11, 979–996. doi: 10.1080/19490976.2020.1732268, 32138583 PMC7524280

[ref55] Tian E. Wang F. Zhao L. Sun Y. Yang J. (2023). The pathogenic role of intestinal flora metabolites in diabetic nephropathy. Front. Physiol. 14:1231621. doi: 10.3389/fphys.2023.1231621, 37469558 PMC10352811

[ref56] Uma A. Sivaraman S. Manoharan R. Periasamy P. (2025). Diabetic kidney disease in type 2 diabetes: a comprehensive review of epidemiology, pathophysiology, and therapeutic advances. J. Pharm. Bioallied Sci. 17, 33–35. doi: 10.4103/jpbs.jpbs_1059_25, 40860001 PMC12373389

[ref57] Vozza A. Volpe S. Custodero C. Colaianni V. Lavarra V. Triggiani D. . (2025). Glucagon-like peptide 1 receptor agonists and sodium-glucose cotransporter 2 inhibitors improve renal resistive index in patients with type 2 diabetes: a 26-week prospective observational real-life study. J. Diabetes Res. 2025:8182211. doi: 10.1155/jdr/8182211, 39963363 PMC11832268

[ref58] Wang X. X. Edelstein M. H. Gafter U. Qiu L. Luo Y. Dobrinskikh E. . (2016). G protein-coupled bile acid receptor TGR5 activation inhibits kidney disease in obesity and diabetes. J. Am. Soc. Nephrol. 27, 1362–1378. doi: 10.1681/ASN.2014121271, 26424786 PMC4849814

[ref59] Wang X. Liu X. Gong F. Jiang Y. Zhang C. Zhou W. . (2025). Targeting gut microbiota for diabetic nephropathy treatment: probiotics, dietary interventions, and fecal microbiota transplantation. Front. Endocrinol. 16:1621968. doi: 10.3389/fendo.2025.1621968, 40661744 PMC12256261

[ref60] Watanabe Y. Fujii H. Aoki K. Kanazawa Y. Miyakawa T. (2009). A cross-sectional survey of chronic kidney disease and diabetic kidney disease in Japanese type 2 diabetic patients at four urban diabetes clinics. Intern. Med. 48, 411–414. doi: 10.2169/internalmedicine.48.1691, 19293538

[ref61] Wu Y. Xu H. Tu X. Gao Z. (2025). Role of metabolic conditions in cardiorenal diseases: initiating pathways and therapeutic targeting. Front. Nutr. 12:1701084. doi: 10.3389/fnut.2025.1701084, 41346672 PMC12672300

[ref62] Yao L. Wang L. Zhang R. Soukas A. A. Wu L. (2025). The direct targets of metformin in diabetes and beyond. Trends Endocrinol. Metab. 36, 364–372. doi: 10.1016/j.tem.2024.07.017, 39227192 PMC12585531

[ref63] Yu Y. Hu G. Yang X. Yin Y. Tong K. Yu R. (2024). A strategic study of acupuncture for diabetic kidney disease based on meta-analysis and data mining. Front. Endocrinol. 15:1273265. doi: 10.3389/fendo.2024.1273265, 38469137 PMC10925656

[ref64] Yu Y. Xu X. Tan D. Yin Y. Yang X. Yu R. (2023). A study on the use of acupoint catgut embedding in the treatment of pre-diabetes: a meta-analysis and data mining approach. Front. Public Health 11:1282720. doi: 10.3389/fpubh.2023.1282720, 38131018 PMC10733528

[ref65] Zhang Q. Lu L. Wang J. Lu M. Liu D. Zhou C. . (2024). Metabolomic profiling reveals the step-wise alteration of bile acid metabolism in patients with diabetic kidney disease. Nutr. Diabetes 14:85. doi: 10.1038/s41387-024-00315-0, 39384774 PMC11464666

[ref66] Zhang Y. Qing J. Saed Y. A. Li Y. (2025). Gut microbiota implication in diabetic kidney disease: mechanisms and novel therapeutic strategies. Ren. Fail. 47:2517402. doi: 10.1080/0886022X.2025.2517402, 40563141 PMC12893496

[ref67] Zhang L. Wang Z. Zhang X. Zhao L. Chu J. Li H. . (2022). Alterations of the gut microbiota in patients with diabetic nephropathy. Microbiol. Spectr. 10:e0032422. doi: 10.1128/spectrum.00324-22, 35863004 PMC9430528

[ref68] Zhao J. Ning X. Liu B. Dong R. Bai M. Sun S. (2021). Specific alterations in gut microbiota in patients with chronic kidney disease: an updated systematic review. Ren. Fail. 43, 102–112. doi: 10.1080/0886022X.2020.1864404, 33406960 PMC7808321

